# Deep learning revealed statistics of the MgO particles dissolution rate in a CaO–Al_2_O_3_–SiO_2_–MgO slag

**DOI:** 10.1038/s41598-024-71640-8

**Published:** 2024-09-11

**Authors:** Fereshteh Falah Chamasemani, Florian Lenzhofer, Roland Brunner

**Affiliations:** 1 Materials Center Leoben Forschung GmbH, Leoben, Styria Austria; 2 Chair of Ceramics, Montanuniversität Leoben, Leoben, Styria Austria

**Keywords:** Refractory, Corrosion, Deep learning, Dissolution, HT-CLSM, Materials science, Techniques and instrumentation, Characterization and analytical techniques, Imaging techniques, Microscopy, Engineering, Computational science

## Abstract

Accelerated material development for refractory ceramics triggers possibilities in context to enhanced energy efficiency for industrial processes. Here, the gathering of comprehensive material data is essential. High temperature-confocal laser scanning microscopy (HT-CLSM) displays a highly suitable in-situ method to study the underlying dissolution kinetics in the slag over time. A major drawback concerns the efficient and accurate processing of the collected image data. Here, we introduce an attention encoder–decoder convolutional neural network enabling the fully automated evaluation of the particle dissolution rate with a precision of 99.1%. The presented approach provides accurate and efficient analysis capabilities with high statistical gain and is highly resilient to image quality changes. The prediction model allows an automated diameter evaluation of the MgO particles' dissolution in the silicate slag for different temperature settings and various HT-CLSM data sets. Moreover, it is not limited to HT-CLSM image data and can be applied to various domains.

## Introduction

Advanced material platforms are underway to accelerate material design in various fields^1–4^. Here, automatized workflows incorporating machine learning for material improvement can lead the way for a transformative reduction of the required development time^2^. A main challenge concerns the establishment of an infrastructure based on FAIR data^5,6^ from all parts of the chain. In order to gather comprehensive material data, characterization methods^7–14^ to study the structure or morphology, are crucial. In particular imaging methods on various length scales represent highly valuable assets for collecting data sets in context to the morphology or structure of the material^8–12,15,16^. In this context, advanced image analysis algorithm capable to treat the collected big data sets time efficiently as well as objectively, are essential rather than a supplement. The extracted structural features can be further correlated with material properties^17–19^ to derive improved material design guidelines^20,21^.

Accelerated material development within the field of refractory materials may also enhance possibilities in context to improved energy efficiency of industrial processes. For the development of refractory materials in particular a fundamental understanding about the dissolution of particles into the molten slag^22–28^ is essential. Refractories often face corrosive wear due to diffusion in liquid slags at high temperatures^29–33^. Therefore, accurate dissolution experiments and efficient quantification approaches are essential for designing refractory products with more wear resistant and long service life. In this context a prerequisite for understanding the dissolution mechanism^13,22,30,34^ is to evaluate the dissolving particle diameter over time. High temperature-confocal laser scanning microscopy (HT-CLSM) represents an important characterization method for the in-situ observation and quantification of the dissolution of micro-particles in the corrosive melt^7,14,22,24,25,27,28,35–40^. Here, it is critical to analyse the dissolving particle images with time in order to acquire the dissolution kinetics^13,41^. This analyses usually involves manually marking or painting particles edges of limited images data from the HT-CLSM experiment^7,13,14,28,42^ and calculating their equivalent diameter using free image processing tools like ImageJ or NIH software^43^. However, the manual method is labour-intensive, time-consuming, subjective, and prone to error due to possible poor image quality, low contrast, camera shaking, shape variation, movement, rotation, and transparency of particles and often very specific in its application. Further, the manual characterization strongly depends on the operator, and hence is problematic for a large amount of data volume or complex image data. Recently, a thresholding approach has been utilized to extract the diameter of the dissolved micro-particles in the slag^44^. Here, a big challenge concerns the correct definition of the threshold based on the grey value distribution. The selection of the threshold is particular demanding when the different material phases show similar grey values and low contrast, artefacts or changes in the particle geometry, as well as brightness variations within the acquired time series.

Deep learning provides intriguing possibilities in the field of image recognition and processing^45^. The U-Net architecture, based on a convolutional neural network (CNN), has become a widely adopted and influential model^46,47^, which can overcome the aforementioned challenges. The modular nature of the U-Net allows for easy customization and adaptability, making it a versatile solution for a wide range of image segmentation problems. In other words, the U-Net architecture represents a cornerstone in image segmentation, providing a powerful and flexible framework that has made significant advancements in various applications^10,12,46–50^. The original U-Net architecture^46^ consists of an encoder and decoder, connected by skip connections. The encoder extracts hierarchical features, while the decoder combines them to create the final segmentation. The attention U-net architecture builds upon the strong foundation of the original U-net architecture by introducing attention mechanisms that allow the model to focus on the most informative features during the decoding process^50–52^. Particularly for complex and challenging medical image segmentation tasks e.g. for tumours, organ, and lesion segmentation^51–53^, aiding disease diagnosis, treatment planning, and monitoring, it could be shown that the attention U-Net outperforms the traditional U-Net approach.

For accelerated material development there is an essential need to develop advanced image analysis tools enabling comprehensive analyses of the HT-CLSM data in a time efficient manner as well as to be suitable for the analysis of various material or micro-particle configurations. Further, the tool shall be independent of the imaging technique and operator inputs.

In this work, we introduce a deep learning-driven workflow for the fully automated evaluation of MgO particle dissolving rates based on in-situ high temperature-confocal laser scanning microscopy (HT-CLSM). For the automated analysis of the image data gathered at different time steps, an attention encoder–decoder convolutional neural network (atU-Net) architecture is utilized to (1) address limitations of manual and thresholding-based evaluations with respect to applicability; (2) provide a multiple-time faster and more accurate evaluation compared to the manual and thresholding-based approach; and (3) deliver sufficient statistical relevant FAIR data which is compatible with accelerated material development. We apply the trained atU-Net on HT-CLSM data obtained from the dissolution of magnesia particles taken from 15 HT-CLSM experiments at three different temperatures with 1450, 1500, and 1550 °C. The additional attention gate within the U-Net architecture can efficiently localise the MgO particles in the slag while boosting overall prediction performance by decreasing miss-segmentation. We reveal that the atU-Net outperforms the conventional U-Net architecture. More precisely, the application of the developed atU-Net is not restricted to the evaluation of particle dissolving rates; rather, it offers an approach for accurate and efficient particle tracking and identification in various data types such as scanning electron microscopy (SEM), micro- X-ray computed tomography (µ-XCT), etc., as well as across other domains.

## Results

### Automated workflow for the particle dissolution analysis over time

**Fig. 1:**
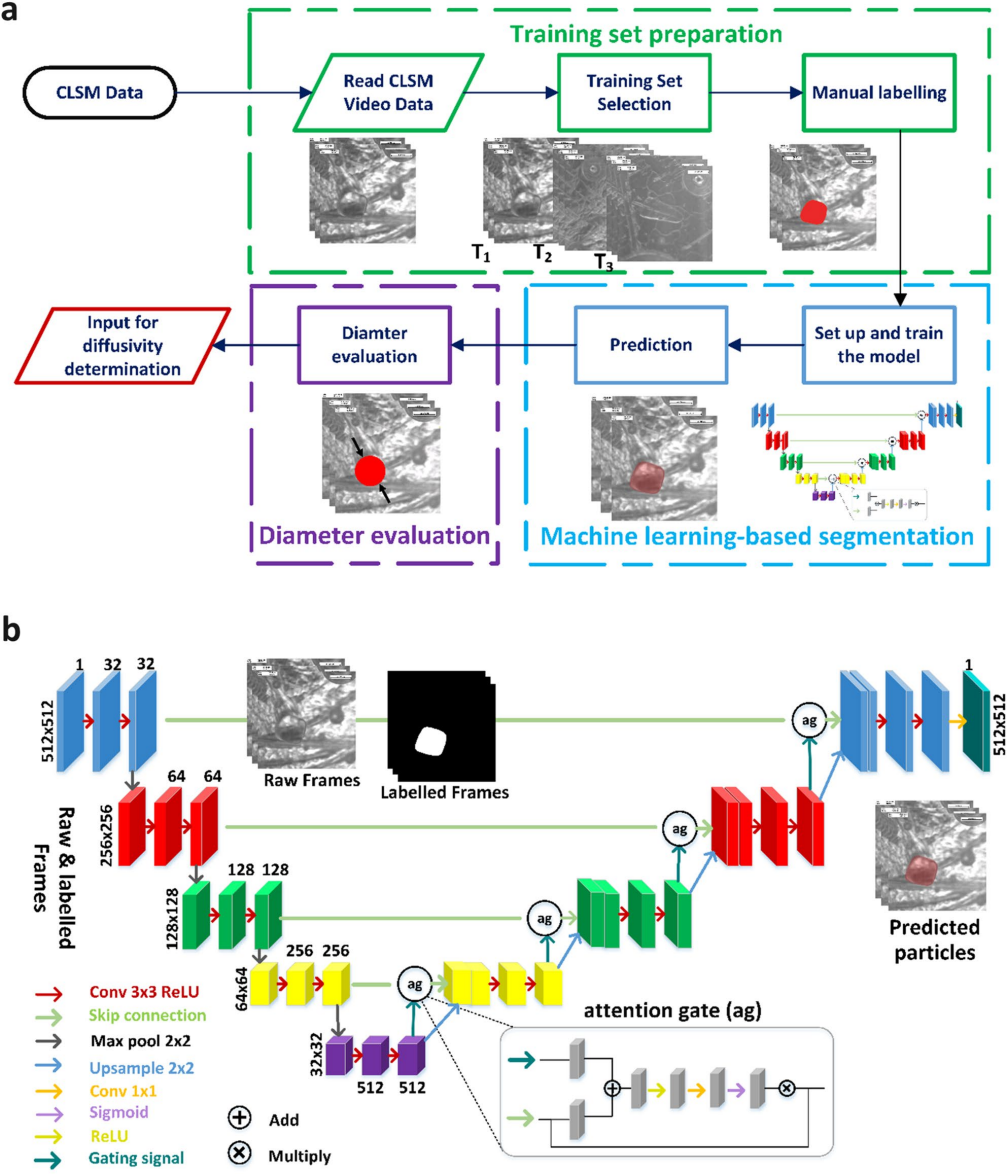
Schematic of the ML-based workflow to quantify the dissolution of the MgO particles within the molten slag utilizing the atU-Net architecture. (**a**) The workflow illustrates three main components indicated by the training set preparation, ML-based segmentation, and particle diameter evaluation. HT-CLSM provides continuously measured image data for defined timeframes, utilized as an input for the analysis workflow. (**b**) The architecture of the developed attention encoder–decoder convolutional neural network (atU-Net) model utilized for the segmentation is illustrated. The input with the raw and labelled frames, various layers as shown in the legend with different colour codings, the attention gates (ag) and output with the predicted particle are highlighted in the schematic. Number of layers are indicated.

The primary focus of the work is to establish an analysis methodology to accurately and efficiently quantify the dissolution of MgO particles with respect to the change of the particle diameter within the molten slag over time. Figure 1a illustrates a schematic representation of the image analysis workflow. The workflow includes the training set preparation for the supervised machine learning (ML) model, as well as the ML-based segmentation, and diameter evaluation of the particles in the molten slag. Image data over time is collected by utilizing a high temperature-confocal laser scanning microscopy (HT-CLSM)-system^34^. With the HT-CLSM the dissolution of the MgO particles within the CaO–Al_2_O_3_–SiO_2_–MgO slag for different time steps can be continuously monitored, see further details in the method section. In this study, 15 HT-CLSM experiments at three distinct temperatures are performed to test the ML-based algorithm. Note, that the algorithm is not limited to this amount of data-set. Here, smaller and larger data sets can be processed in a straightforward manner.

The training set preparation, highlighted by the green box in Fig. 1a, includes the import of the generated raw HT-CLSM video data, the training set selection, and the manual labelling process, necessary to generate the training data set. These three processes are crucial to set up and train the deep learning atU-Net model, sufficiently. We train the atU-Net model on the particle images (raw frames) and their corresponding labelled/annotated images using 7% of all raw frames obtained from 15 HT-CLSM videos. The initial steps involve 30% of the training set and its augmentation with basic image manipulation, to increase robustness and generalization while minimizing the risk of overfitting, see further details in the method section and Supplementary Fig. 1. Figure 1b shows the architecture of the developed atU-Net consisting of an encoder path (on the left) and a decoder path (on the right) and, in between, the concatenation of feature maps that provide the localization information, see further details in the Method section. Subsequently, the trained atU-Net model is applied to the test sets, and the particles are segmented accordingly. Within the final step the equivalent diameter of the segmented particle for the different time steps is calculated, see Supplementary Note 1.

### Attention encoder–decoder-Unet (atU-Net)-based particle prediction

**Fig. 2:**
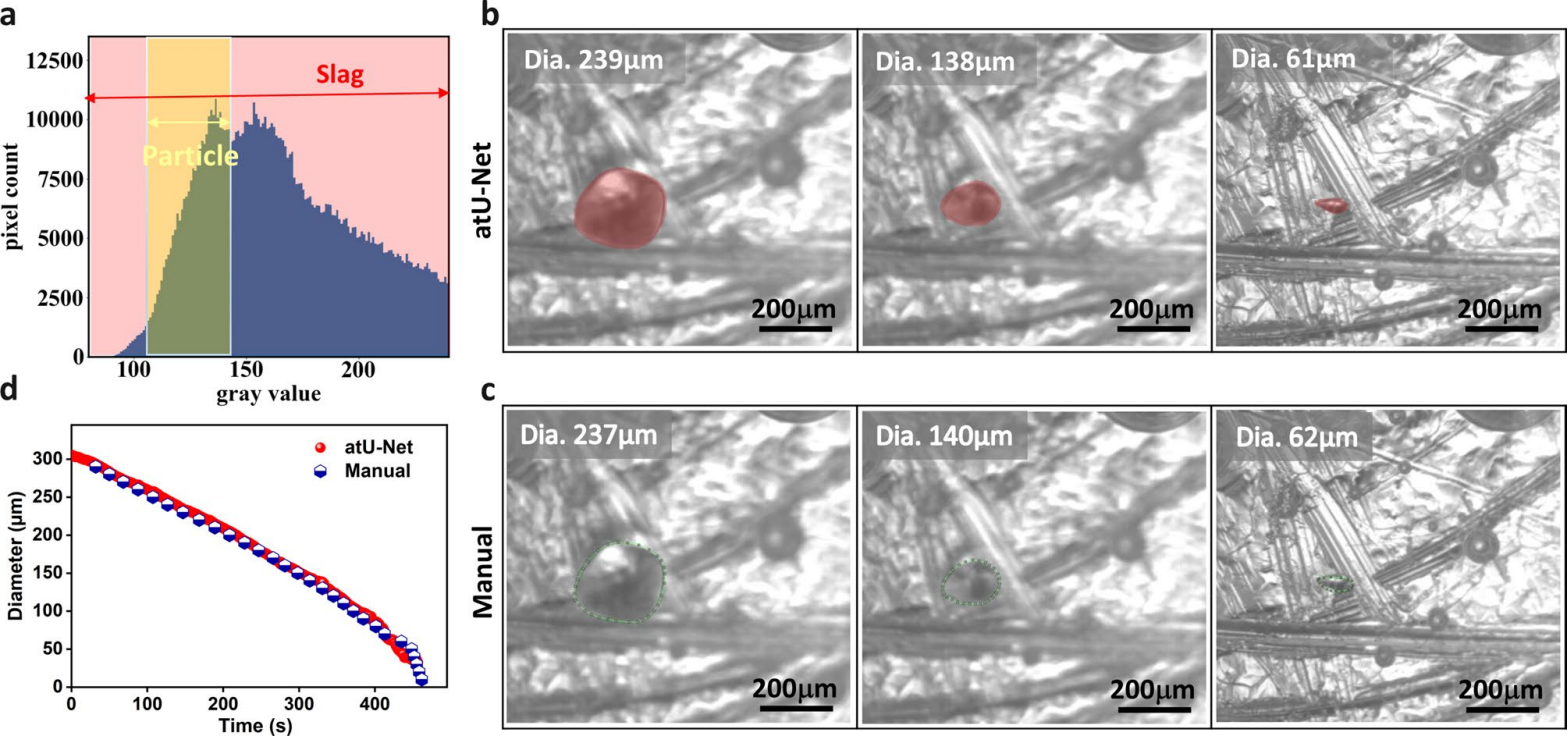
The atU-Net particle prediction accuracy for an exemplary video data-set at 1450 °C. (**a**) Grayscale histogram depicts the intensity area of the MgO-particle (yellow) and the slag (pink) for this selected video. (**b**) Three representative dissolution times that qualitatively show the accuracy of particle segmentation utilizing the developed atU-Net model. The segmented MgO-particle attained from the atU-net segmentation are highlighted in red. The abbreviation “Dia.” stands for particle diameter. (**c**) Illustrates the manual evaluation for the same dissolution time. The particles are indicated by the manually drawn particle boundary in green. (**d**) The evaluated diameter evolution over time is shown for the deep learning atU-Net model (red dots) and the manual evaluation (blue dots), with 1398- and 32-time-steps, respectively.

Figure 2a indicates the grayscale histogram for the intensity area of the particle (yellow) and the slag (pink) exemplarily. Challenging is the segmentation of the particles from the slag since the material phases show similar grey values. Therefore, a simple thresholding approach is demanding and an accurate segmentation will rather fail. As shown in Fig. 2b, the developed atU-Net model is capable to perform an accurate segmentation. Segmented MgO-particles at three different time stages of the dissolution (early, middle, and near the end) are presented in Fig. 2b from left to right, respectively. Figure 2b indicates the segmented particles highlighted in red utilizing the atU-Net model. The manual segmentation is illustrated in Fig. 2c. Here, the MgO-particle edge (boundary) is indicated by a dotted line in green. Further, we show in Fig. 2d, the extracted MgO-particle diameter over time, conducting the atU-Net model and the manual analysis. With the atU-Net, the particle diameter from the HT-CLSM data for 1398-time steps can be derived within less than five minutes. Contrary to the manual segmentation utilizing ImageJ, where only 32 selected time steps can be extracted within a timeframe of hours. Note, that the manual analysis of the full dataset is too costly with respect to the necessary time effort. The calculated precision of the atU-Net provides 99.1% indicating a high accuracy although the two material phases of the MgO-particle and CaO–Al_2_O_3_–SiO_2_–MgO slag show similar grey values and small contrast within the collected image data.

### Resilience of the developed atU-Net with respect to contrast and shape

**Fig. 3:**
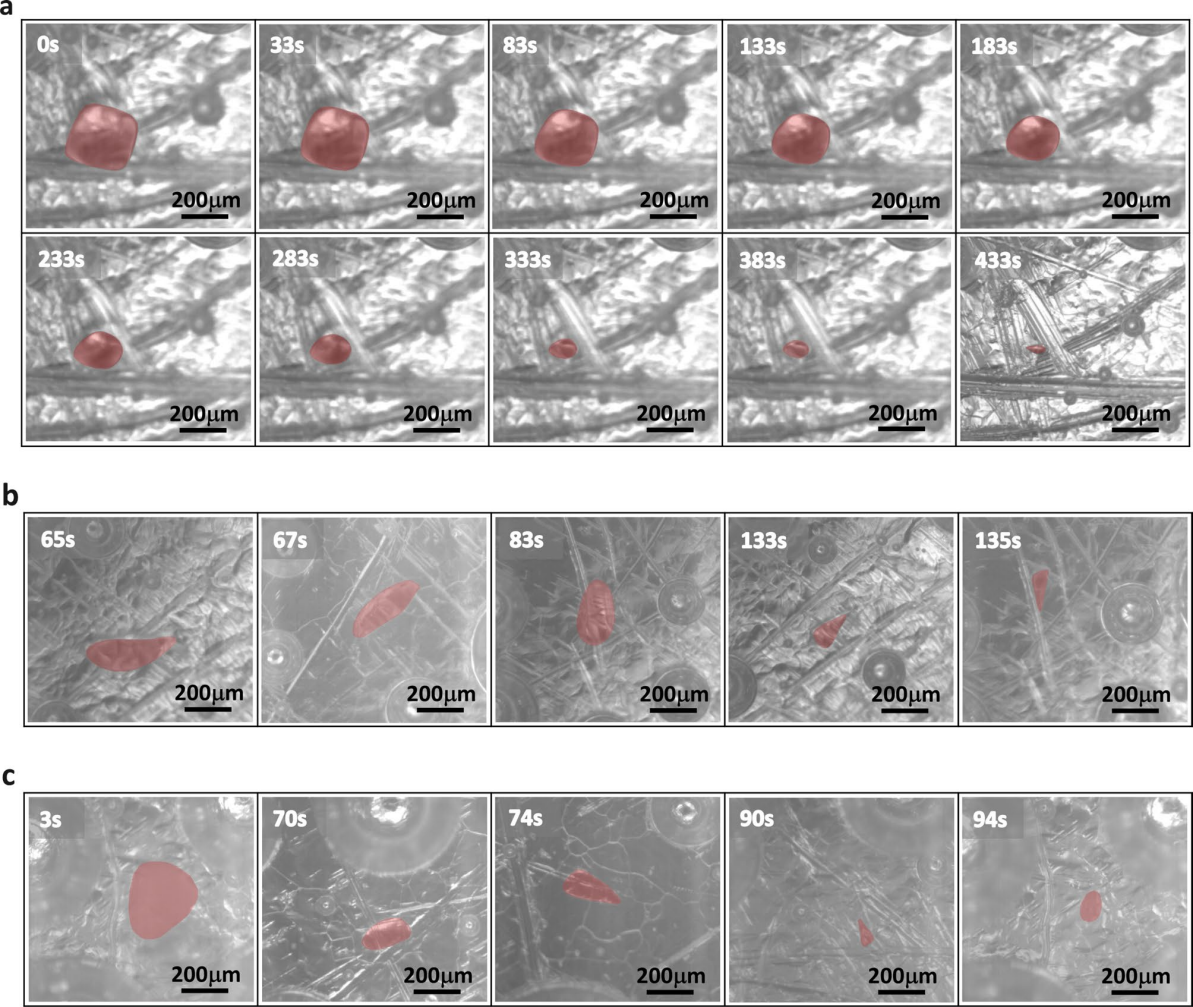
atU-Net MgO-particle prediction in context to the diversity of particle shape, contrast and brightness for different temperatures. (**a**) Depicts the MgO-particle prediction from the atU-Net for ten dissolution time-steps along with the evaluated diameters at T = 1450 °C. A rather spherical-like shape is depicted. (**b**) MgO-Particle prediction at T = 1500 °C, with a rather elongated spherical-like shape. (**c**) Particle prediction at T = 1550 °C with a rather rectangular-like shape. In addition, changes in contrast and brightness are illustrated for the different frames. The predicted particles are highlighted in red and indicate the actual MgO-particle. The image acquisition or dissolution time is shown in the top left of the images. The scale bar is 200 µm for all images.

In the following, we assess how the inhomogeneous image quality affects the atU-Net model. Figure 3a, shows exemplarily the MgO-particle diameter prediction for ten time-steps conducting the atU-Net model at a defined temperature at T = 1450 °C. Qualitatively, it can be shown that the quasi-spherical particles are well segmented. At 1450 °C the shape of the particles is not changing significantly with time and the semi-spherical shape is conserved. Note, that the particle shape changes once the temperature is increased to 1500 °C or even 1550 °C, see Fig. 3b and c, respectively. Here, elongated spherical as well as rectangular-like particle shapes of the MgO-particle appear during the dissolution process within the molten slag. In addition, for the different frames variations in contrast and brightness emerge, see for example Fig. 3b. Yet, as illustrated in Fig.3 the developed atU-Net is resilient to the shape of the particles, the image contrast between the slag and particles, as well as brightness changes caused by temperature or the proximity of the particle to the crucible.

### Statistical analysis of the particle dissolution in the molten slag

**Fig. 4:**
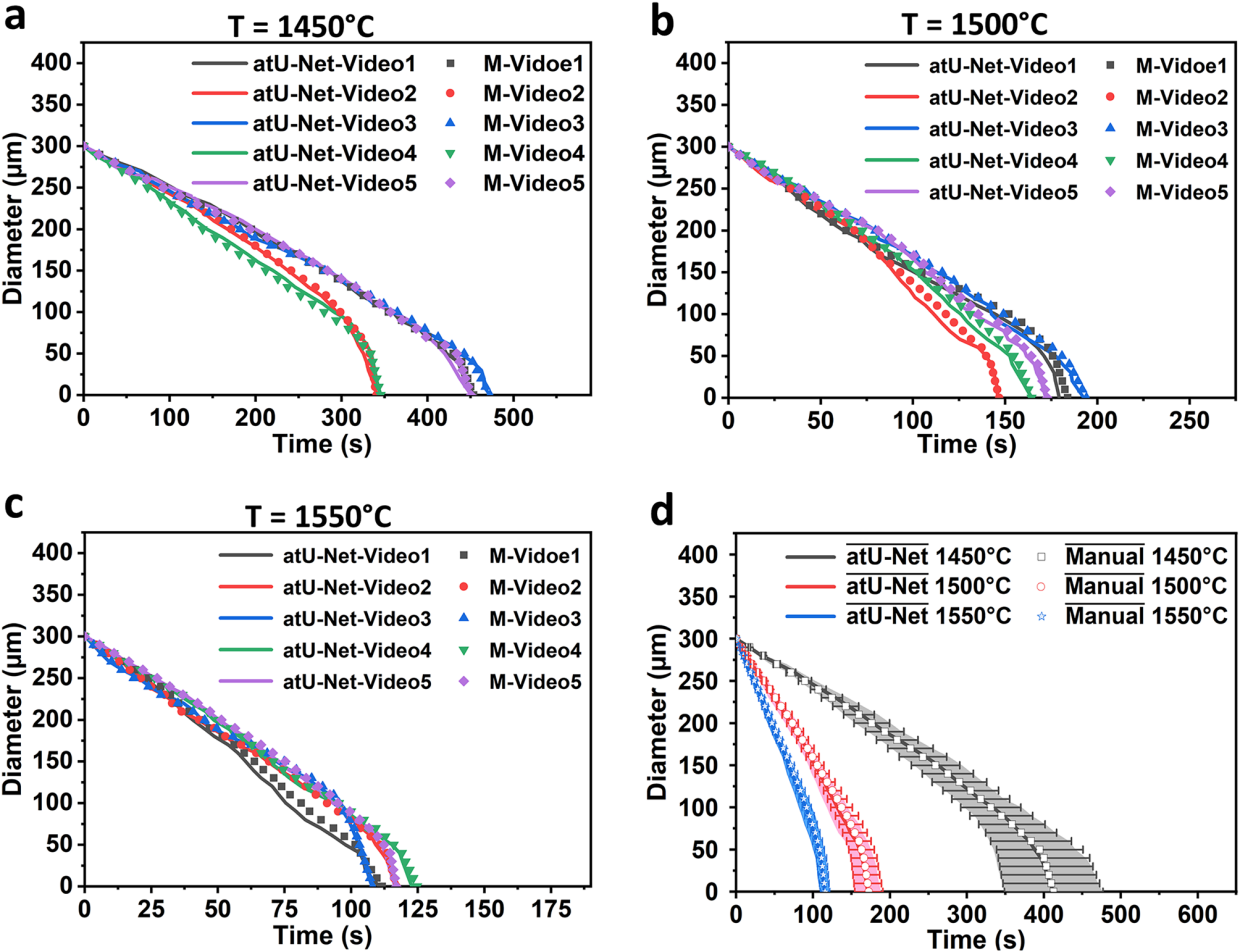
Statistical analysis of the particle diameter with time at three different temperatures and different video data sets. At (**a**) 1450 °C, (**b**) 1500 °C, and (**c**) 1550 °C five different video data sets are analysed to understand the particle dissolution over time. The atU-Net prediction for the different videos is indicated as atU-Net-Video 1–5 and for the manual evaluation as M-Video 1–5. The five different videos for each temperature are indicated by different colors (dark grey, red, blue, green and purple). (**d**) Diagram which shows the mean MgO-particle diameters extracted from the five video data-sets, utilizing the atU-Net prediction and the manual evaluation (indicated with $\overline{\text{atU}-\text{Net}}$ and $\overline{\text{Manual}}$, respectively) at each temperature. The shaded area and the error bars around the mean diameters indicate the standard deviation of the atU-net prediction and the manual evaluation, respectively. The results show a reduction in the particle's dissolution time as the temperature increases, as well as good agreement between atU-Net and manual evaluation.

Figure 4, indicates the statistical analysis based on the developed atU-Net. For a better comparison of the collected different datasets the evaluation begins with an equivalent diameter of 300 µm. Then, the trend function is applied to evaluate  the change of the related diameter versus the dissolution time. We evaluate the prediction by using the manual evaluation as a ground truth. For all fifteen videos at the three experimental temperatures of 1450, 1500, and 1550 °C a sufficient resemblance is indicated in Fig. 4a–c. The deviation between the atU-Net prediction and the manual analysis is about 1%. This deviation is mainly caused in the manual evaluation by an overestimation of the particles' edges during manual boundary marking, using the segmented line tool in ImageJ for particle identification. In addition, we show in Fig. 4d the computed mean and standard deviation of the obtained diameter for all five videos at each experimental temperature over time for the atU-Net prediction (shaded areas around the mean diameter). Clearly, the results indicate that the MgO-particle's dissolving time increases with decreasing temperature. The atU-Net model clearly allows an automatized and efficient analysis of the whole data set depicted from the HT-CLSM in-situ experiment. A comparison of the atU-Net with a conventional U-Net is illustrated in Supplementary Figs. 2–4. The prediction results obtained from the atU-Net show better agreement with the manual evaluation compared to the conventional U-Net, see Supplementary Note 2 and Supplementary Fig. 5).

## Conclusion

Accurate, time-efficient and statistically relevant quantification of the particle dissolution within the molten slag represents an essential ingredient for the accelerated development of refractory materials which requires comprehensive material data, in context to extending the service life, reducing wear, and improving energy efficiency in industrial processes. Within this study, we develop a deep learning approach to analyse the particle dissolution in a molten slag using image data obtained from in-situ HT-CLSM. We monitor the dissolution of MgO-particles within a silicate slag exemplarily at three different temperatures. Such HT-CLSM video data-sets are usually analysed either manually or by image thresholding. Nevertheless, both methods are insufficient for a comprehensive statistical relevant study due to their time-consuming nature and necessary user expertise, as well as their restricted application and inability to adapt to changes in particle geometry and image quality. The state-of- the-art image analysis methods often make extensive re-development necessary to meet different sample criteria. In this study, we employ an attention encoder–decoder, atU-Net model, to accurately and efficiently quantify the MgO-particle dissolution in the molten slag. We show that the developed deep learning model is highly resilient to changes in context to shape, brightness or contrast. It demonstrates the successful prediction of low-contrast and blurry particle images or frames. The atU-Net prediction for the particle diameters in different dissolution experiments at three different experimental temperatures provides a precision of 99.1%, indicating high accuracy. Moreover, the model is not restricted to the material under investigation but can be also applied to particle tracking and identification in various domains, as well as to different imaging methods.

## Methods

### Sample preparation

In this research work, fused magnesia particles of 300–500 µm with a magnesia content of 98.66 wt% and a density of 3.58 g/cc have been studied in a silicate slag with CaO/SiO_2_ (C/S) weight ratio of 1.17. The synthetic silicate slag in the system CaO–Al_2_O_3_–SiO_2_–MgO (CASM) was produced from decarburized calcium carbonate, alumina powder, quartz powder, and magnesia powder (S3 Handel und Dienstleistungen UG). All the raw materials were weighed as per target slag composition and mixed to prepare the slag batch. To enhance the homogeneity, the slag batch was molten in a platinum crucible in a preheated furnace at 1450 °C for 15 min and quenched on a steel plate. Further to enhance homogeneity and to facilitate its application (to maintain same slag quantity in all HT-CLSM crucibles), the solidified quenched slag was powdered in a tungsten carbide lined cup mill. In two steps (0.08 g + 0.12 g in each crucible), powder slag was pre-molten in platinum-10% Rhodium (Pt-Rh10) crucibles (frustum shaped cones with upper and lower diameters of 7.5 mm and 6.09 mm, respectively, and a height of 4 mm) at 1450 °C for 15 min in a preheated furnace for easy removal of bubble which is detrimental in HT-CLSM dissolution experiment. The composition and the slag properties, dynamic viscosity ($\eta$), density (${\rho }^{l}$), and liquidus temperature (${T}_{L}$) are tabulated in Table 1.

**Table 1:** Slag properties.

Slag	CaO [wt%]	Al_2_O_3_ [wt%]	SiO_2_ [wt%]	MgO [wt%]	*η*_1450 °C_ [Pas]	*η*_1500 °C_ [Pas]	*η*_1550 °C_ [Pas]	*ρ*_1550 °C_ [kg/m^3^]	*T*_*L*_[°C]
CASM slag, C/S = 1.17	45.03	11.33	38.64	5.00	0.36	0.27	0.20	2653	1317

### Experimental setup

The dissolution experiments of magnesia real particles at 1450, 1500 and 1550 °C in ambient atmosphere were carried out with HT-CLSM. A detailed description of the HT-CLSM device used for the present work can be found in^42,54^. The experimental procedure reported by Harmuth and Burhanuddin^34^ was adopted for this research work. As the furnace thermocouple is situated a few mm below the slag-filled crucible, the measured furnace temperature may differ from the slag temperature. The offset of both may change when the halogen bulb is replaced, as it also depends on the filament position. Therefore, before starting the dissolution experiments, the relation between furnace and sample temperature was always investigated. The sample temperature was measured using an S-type thermocouple embedded in the slag and connected to a data logger, and the difference was incorporated into the temperature schedule to actually achieve the desired slag temperature. At room temperature, the particle was dropped at the center of the Pt-Rh10 crucible within the pre-molten slag with the help of a forceps. Then the mirror furnace was heated to 150 °C at a rate of 50 °C per minute, after 1 min holding at 150 °C heating rate was first increased to 500 °C per minute until 50 °C below the experimental temperature and then decreased again to 100 °C per minute to achieve the further temperature rise and to avoid overheating. Automatic temperature control was kept on hold and manually controlled to avoid overheating and to maintain the experimental temperature until complete dissolution of the particle and then the furnace was cooled at a rate of 400 °C per minute^34^. The Video was captured from 150 °C until the whole dissolution process at the experimental temperature proceeded. Particle tracking, camera focus, and brightness adjustment were controlled manually. Five experiments were carried out at each experimental temperature to check the repeatability and get an average result.

### Conventional image processing

In our early systematic attempts towards an automatized analysis of the dissolving particles, we deploy three conventional image segmentation techniques: intensity-based (thresholding^11^); active contour^55^; and graph-based (Felzenszwalb^56^) to distinguish (segment) the particles from the slag. First, to enhance the data quality, the contrast was enhanced and the noise-level was decreased. Then, one of the above mentioned segmentation methods was applied. In the next step, the segmentation errors were fixed by applying the morphological operation of erosion. Finally, the equivalent diameter of the segmented particles was calculated for possible subsequent analysis like the diffusivity calculation. The presented segmentation techniques are applicable based on particle and slag properties and composition. The first technique was appropriate for the segmentation of zirconia particles, which are not transparent and have a few movements, see Supplementary Note 3 and Supplementary Fig. 7. This technique failed for an accurate segmention of the particle in each dissolution time-step due to intensity inhomogeneities within the image data. An example for the failed segmentation is shown in Supplementary Fig. 8. The active contour technique was used to optimize the segmentation accuracy of the intensity-based technique (Supplementary Fig. 8 and 9).  

The above mentioned two methods are not applicable for the evaluation of the dissolution of sapphire particles. The sapphire particles are transparent and moving around in the crucible. Due to the particle movement, the image data is getting blurred. The particle transparency makes the background (slag) visible through the particle, which makes the segmentation more problematic and critical when using the first two segmentation techniques. The graph-based (Felzenszwalb) method is a solution to tackle these challenge. Although this method is able to accurately segment the sapphire particle (Supplementary Fig. 10) in some experimental HT-CLSM data, its parameter tuning is very tough, and cluster refinement is time-consuming, as shown in Supplementary Fig. 11 and explained in Supplementary Note 4.

Although these techniques succeed in obtaining results for zirconia and sapphire, they failed in the evaluation of magnesia dissolution data due to the following challenges: (a) Discrimination between the particles and the surrounding area (i.e. slag) in terms of intensity (grey value) is almost impossible since their intensity range overlaps (yellow and pink shaded areas in the histogram of the exemplary particle image in Fig. 2a. (b) Particles’ edges are not sharp enough. (c) Diversity of particle behaviours in terms of transparency and movement, which leads to low illumination, contrast insufficiency, and shape diversity over time and different temperatures, as well as blurriness of particles and slags due to the particles' movement. Hence, the application of conventional image segmentation methods like intensity-based, active contour, graph-based, and edge detection^44^ on HT-CLSM data are data dependent and cannot be generalized.

### atU-Net model structure and training

Figure 1b shows the architecture of the developed atU-Net, which includes the encoder and decoder paths that are connected by a bottleneck or bridge. The architecture of the encoder path is based on the common convolutional network^46^. In each step of the encoder path, there are two convolutions with a 3 × 3 kernel size, a rectified linear unit (ReLU) as an activation, and the same padding to cover the boundaries of the input image and have the same output dimension. After the first convolution, there is a dropout layer for avoiding overfitting, and following the second convolution, the pooling layer (max pooling with 2 × 2 operation) is responsible for down-sampling (reduction of the spatial dimension and doubling the feature map). The middle layer (after the encoder path) consists of the two convolution layers and a dropout layer with the same settings as the encoder path. In the decoder path, every step comprises an attention gate and a transposed convolution layer (with a kernel size of 2 × 2, and the same padding) for up-sampling, followed by a skip connection. The long skip connection combines or concatenates the feature map from the transposed layer with the associated one from the encoder path in order to recover spatial information lost during down-sampling. The rest of the steps are similar to the encoder path, which includes two convolutions and a dropout in between. The final layer is a 1 × 1 convolution with a sigmoid as an activation. Supplementary Note 2 and Supplementary Fig. 13 show the structure of the developed U-Net model.

The attention gate (ag) takes inputs of up-sampling and of skip connection, with an emphasis on more feature and spatial information, respectively. A 1 × 1 convolution layer with strides of 1 and 2 is applied to compress the dimensions and make the equal dimension for element-wise summation. This mechanism causes aligned weights to get larger and unaligned weights to shrink. The generated vector passes through a ReLU activation layer. Then, the vector is scaled using a sigmoid layer, resulting in attention coefficients, with weights closer to 1 indicating more significant information. Following resampling, this output is multiplied element-wise by the skip connection input.

We train the atU-Net model on the particle images (raw frames) and their corresponding labelled/annotated images (see Supplementary Note 5 and Supplementary Fig. 12) using 7% of all raw frames obtained from 15 HT-CLSM videos. The initial steps involve training with 30% of the training set and its augmentation with basic image manipulation to increase robustness and generalization, while minimizing the risk of overfitting. The augmentation includes randomly rescaling the images and rotating them by an angle of 90°, 180°, or 270°. The network is trained over a combination of hyperparameters and evaluated on the test set. The best performance of the model is achieved using the Adam optimizer and binary cross entropy as the loss function with a batch size of 10 on image size of 512 × 512 pixels. The model is saved to be retrained with the remaining images (70%) from the training set. After 100 epochs, the trained atU-Net model shows good accuracy and loss values (Supplementary Fig. 1). The trained model is then applied to all the data sets.

We perform all tasks of the training set preparation using Python libraries such as Numpy, Scipy, Scikit image and Ilastik^57^ for manual annotation or labelling. TensorFlow 2.2.0, Python 3.7, and Python libraries’ Numpy, Scipy, Scikit image, and Scikit learn are used to develop and train the atU-Net model. Note, that currently, the model sometimes fails to perfectly segment particles smaller than 30 µm since, near the end of dissolution, the particles are fading in the slag, and separating particles and slag is truly hard. However, this is also challenging for a manual analysis. Moreover, there is a lack of labelled images with particle sizes below 50 µm.

## Supplementary Information

Supplementary Information.

## Data Availability

All data that support the findings of this study are available from the corresponding author upon reasonable request.

## Code availability

All code that support the findings of this study are available from the corresponding author upon reasonable request.
