# Supplementary material for: Deep learning revealed statistics of the MgO particles dissolution rate in a CaO–Al2O3–SiO2–MgO slag
Source: Sci Rep. 2024 Sep 11;14:21279. doi: 10.1038/s41598-024-71640-8 (PMC11390962; doi:10.1038/s41598-024-71640-8)
Supplement: Supplementary file 1 — Supplementary Information. [file 41598_2024_71640_MOESM1_ESM.docx]

**Supplementary Information**

Deep Learning revealed statistics of the MgO particles dissolution rate in a CaO–Al_2_O_3_–SiO_2_–MgO slag

Fereshteh Falah Chamasemani^1^, Florian Lenzhofer^1,2^, Roland Brunner^1,*^

*Corresponding author: [roland.brunner@mcl.at](mailto:roland.brunner@mcl.at)

^1^ Materials Center Leoben Forschung GmbH, Leoben, Styria, Austria

^2^ Chair of Ceramics, Montanuniversität Leoben, Leoben, Styria, Austria

**SUPPLEMENTARY FIGURES**

**
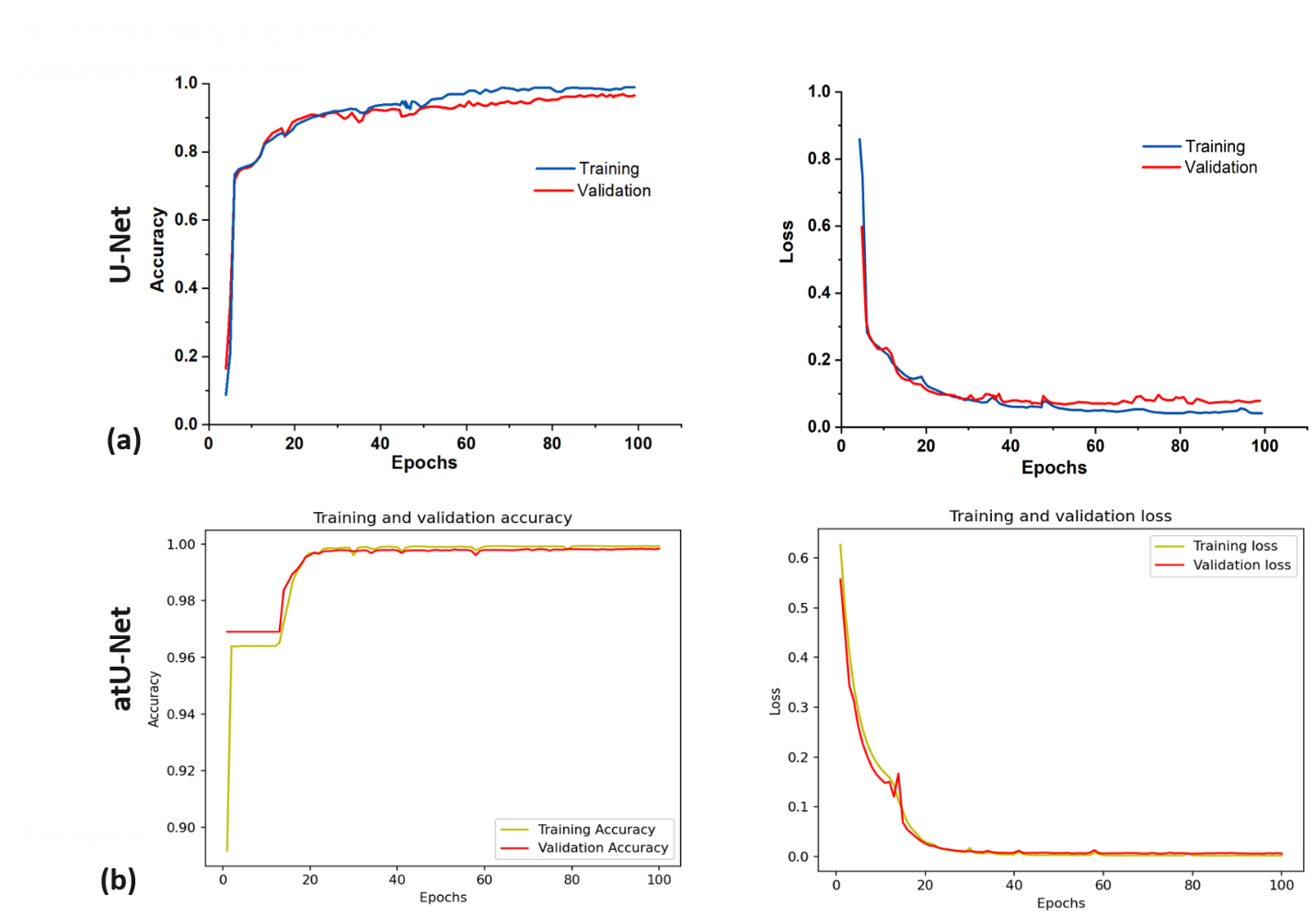
**

**Supplementary Figure 1|** **Accuracy and loss plots of the trained models.** Here, we show the training and validation accuracy and loss curves for the **(a)** U-Net model and **(b)** the atU-Net.


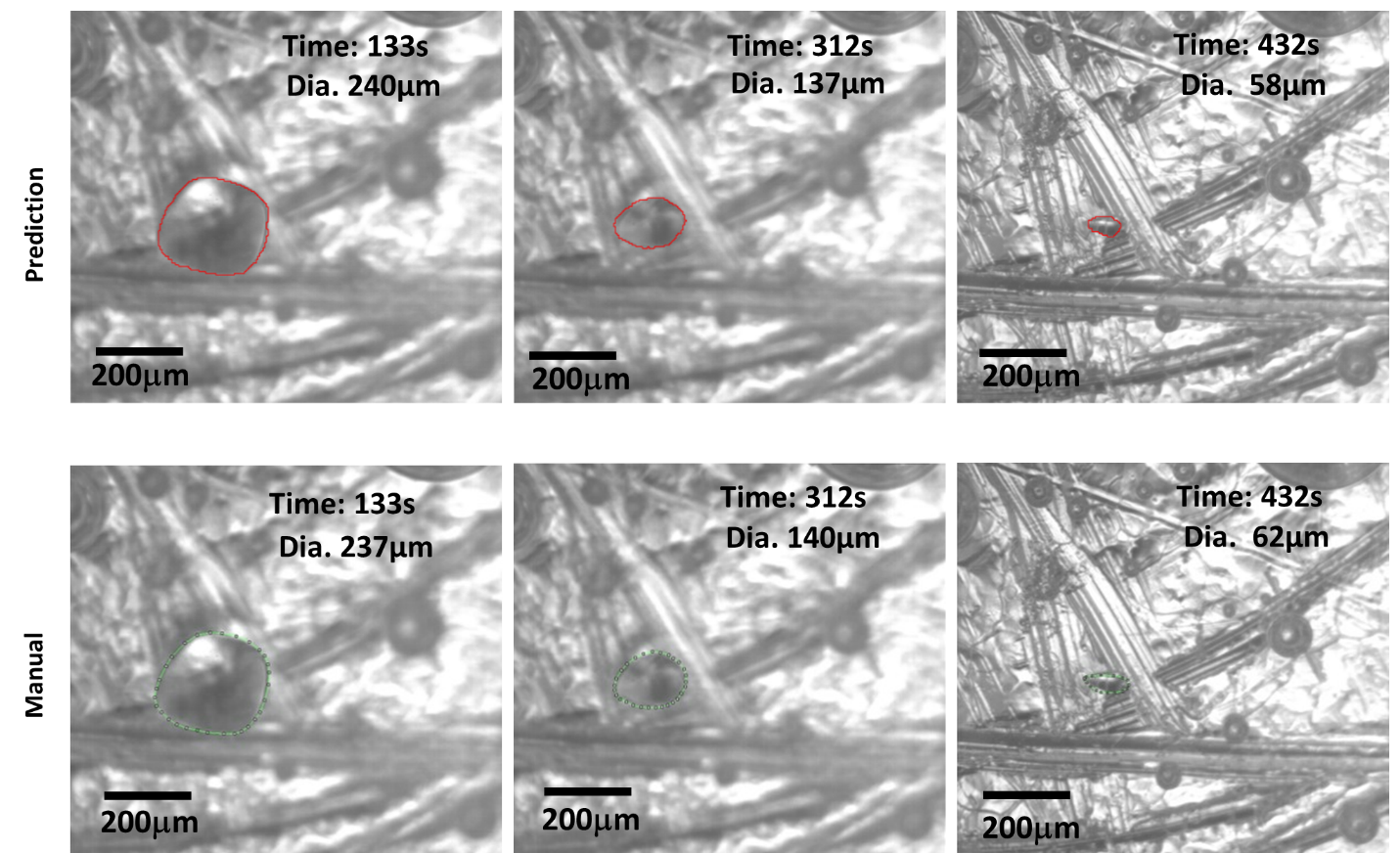


**Supplementary Figure 2|** **The U-Net particle prediction accuracy for an exemplary video at 1450°C.** Three representative dissolution times that qualitatively show the accuracy of particle prediction obtained from the developed U-Net. The red lines highlight the particle boundary/edge attained from the U-Net segmentation. “Dia.” stands for particle diameter. Below we illustrate the manual evaluation of the particles. The particles are indicated by the manually drawn particle boundary in green.


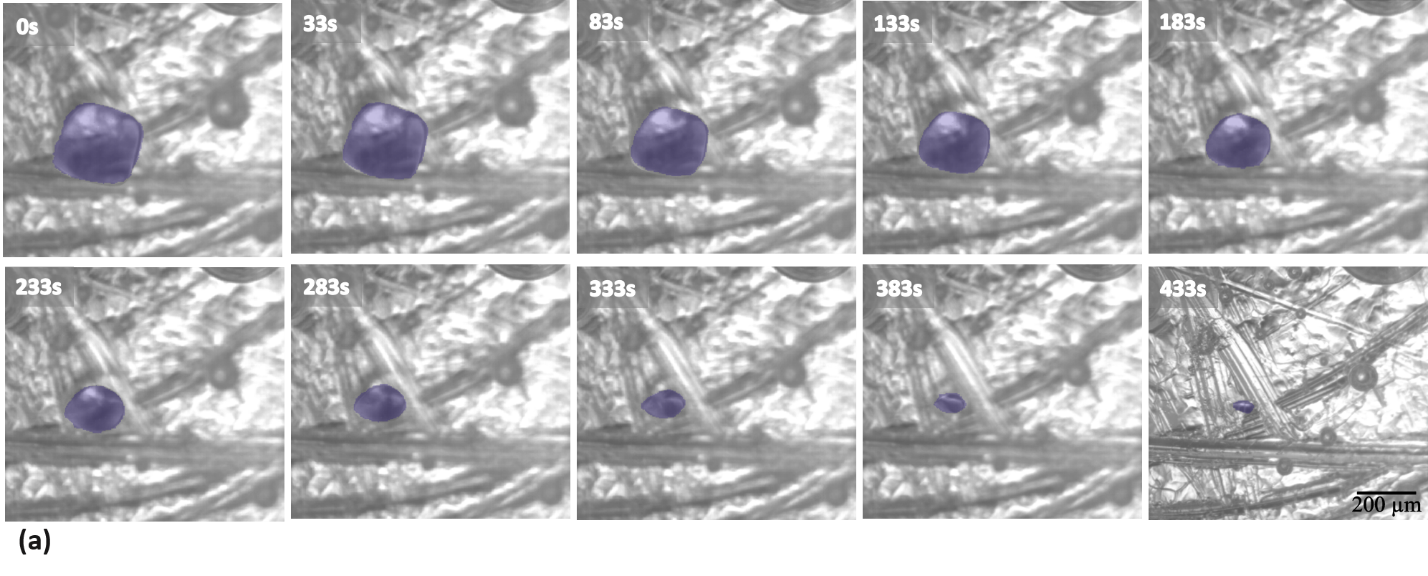


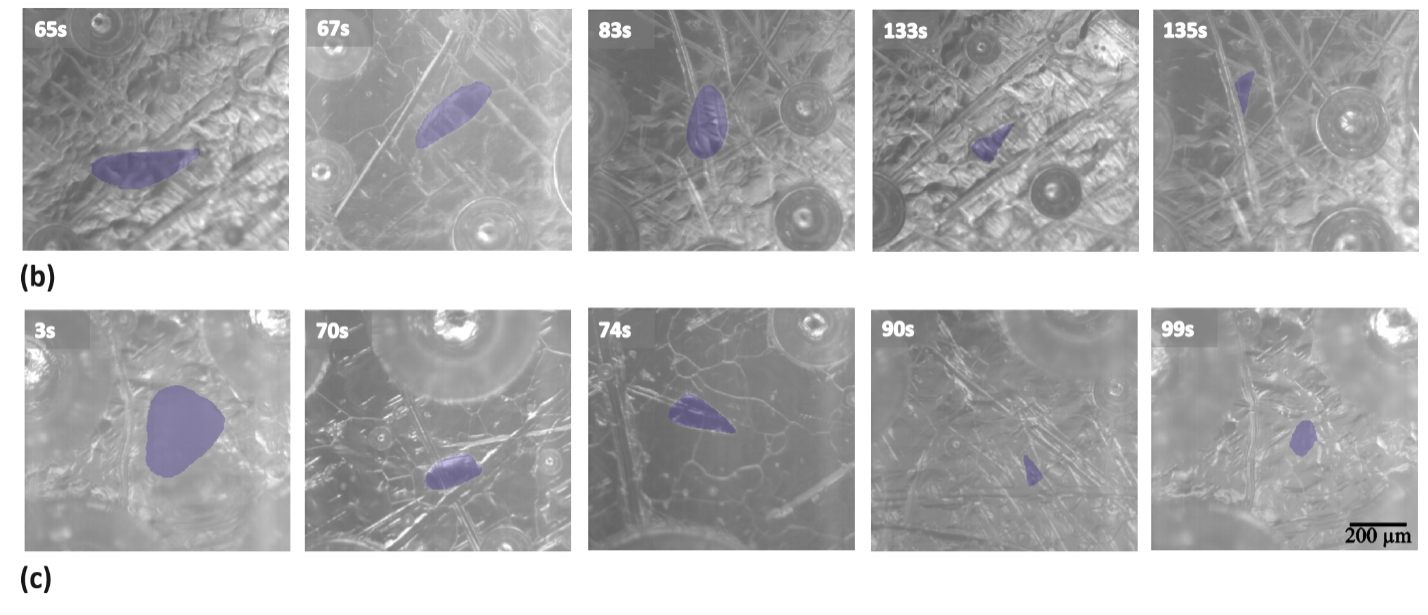


**Supplementary Figure 3| U-Net particle prediction in context to diversity of particle shape, contrast and brightness.** **(a)** depicts the particle prediction from the U-Net for eight dissolution time steps along with the evaluated diameters at T=1450°C. With a rather spherical-like shape. **(b)** Particle prediction at T=1500°C, with a rather elongated-like shape. **(c)** Particle prediction at T=1550°C with a rather rectangular -like shape. The scale bar of 200 µm is indicated on the bottom right side and is valid for all images. The yellow and red lines around the particles show the particles boundary predicted by the U-Net and elaborate on the good accuracy of the prediction. The temperature and time of the experiments are shown in the top right of the images.


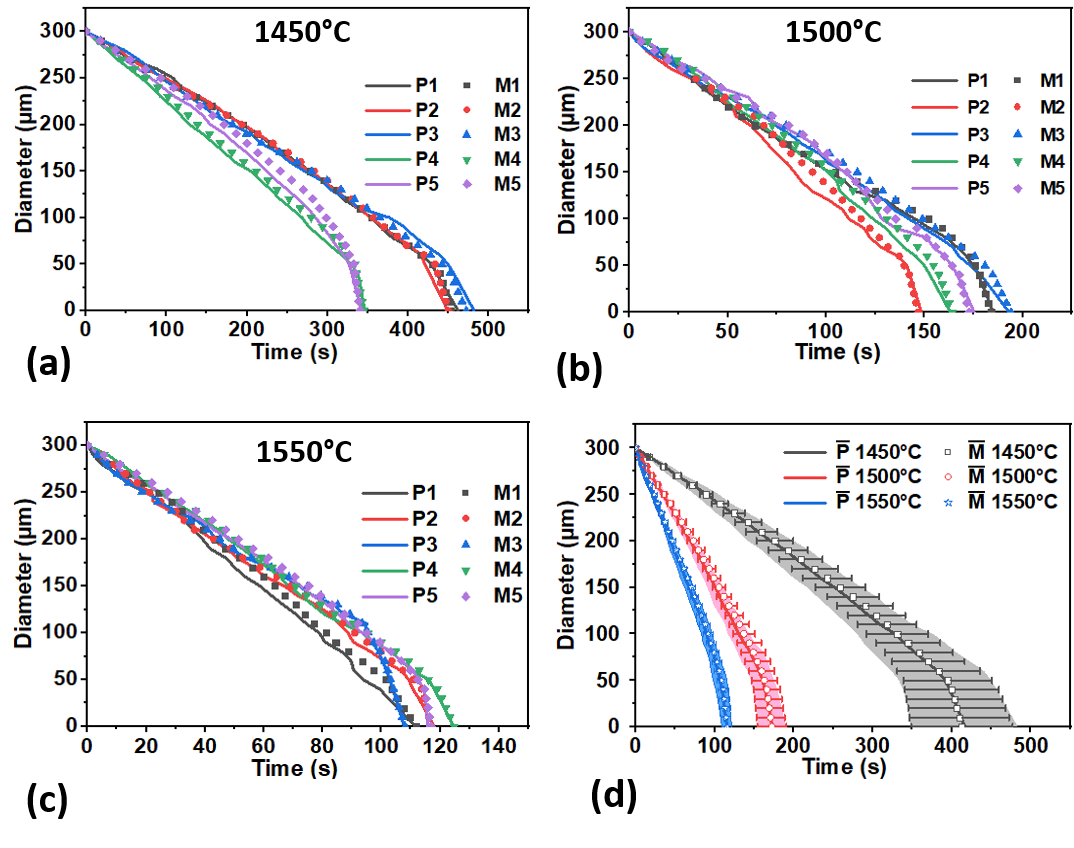


**Supplementary Figure 4|. Statistical analysis of the particle diameter with time at three different temperatures for the U-Net. (a)-(c)** the trend-based comparisons of the particle diameter over dissolution time between the U-Net model prediction (P) and the manual evaluation (M) for all five videos (1–5) at 1450°C, 1500°C, and 1550 °C. Different colors show individual evaluations for each experiment. (d) shows a comparison diagram of the mean diameters (of five experiments) obtained from the U-Net prediction and the manual evaluation (indicating with $\overline{P}$ and $\overline{M}$, respectively) at each temperature and their corresponding standard deviations. The shaded area and the error bars around the mean diameters show the standard deviation of the U-Net prediction and the manual evaluation, respectively. The results show a decrease in the particle's dissolution time as the temperature increases.


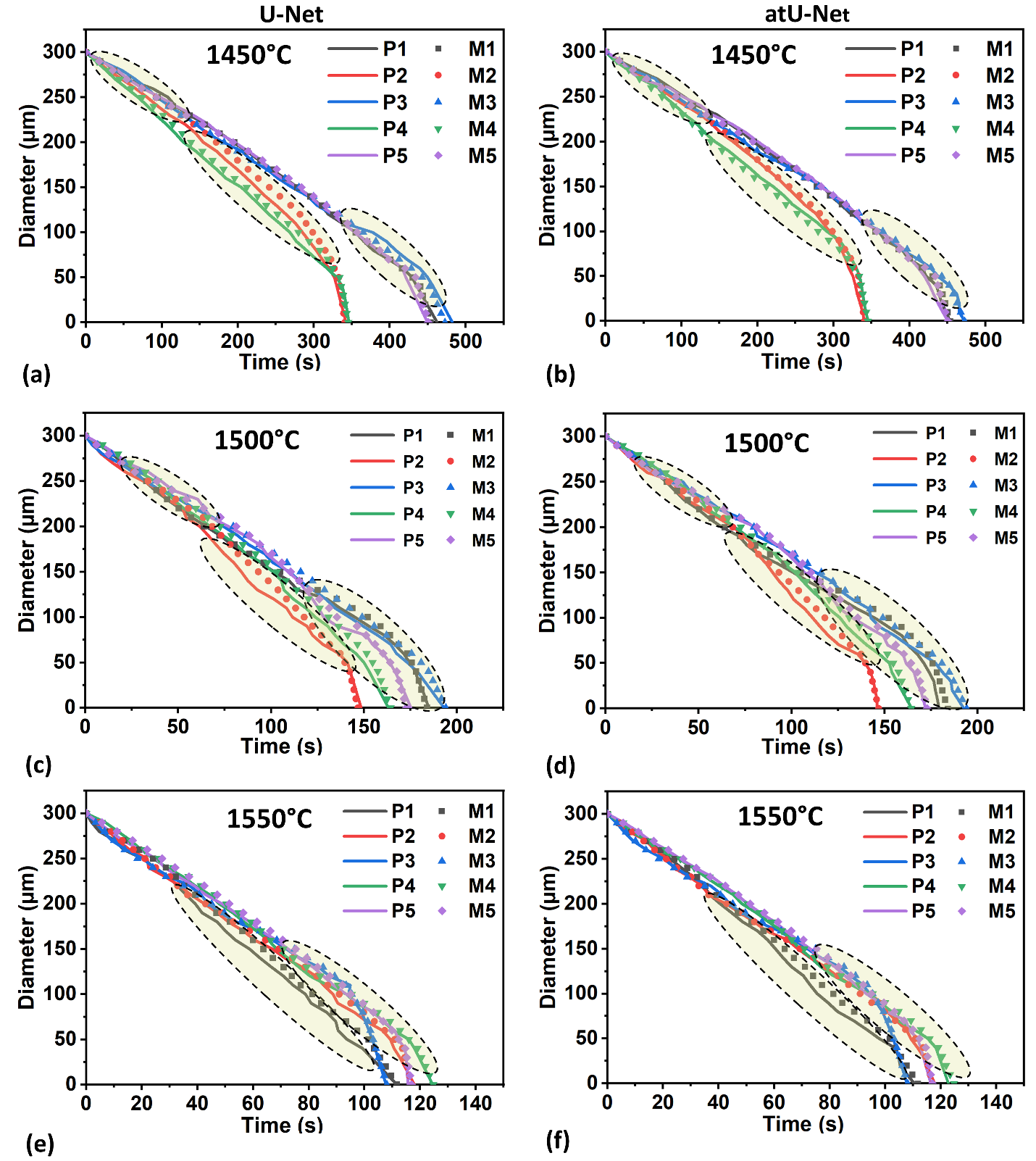


**Supplementary Figure 5|.** **Comparison of atU-Net and U-Net for the particle diameter with time at three different temperatures.** Comparisons of U-Net and atU-Net for **(a)-(b)** 1450°C, **(c)-(d)** 1500°C and **(e)-(f)** 1550°C. The particle segmentation is performed with the prediction model (P) and the manual evaluation (M) for all five videos (1–5). Deviations are highlighted with dashed lines.

**
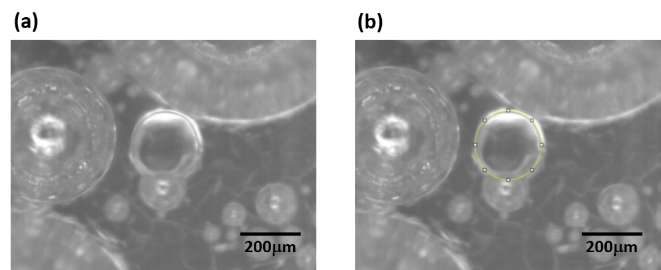
**

**Supplementary Figure 6|** **Manual dissolution tracking of a sapphire particle in a CAS slag (CaO, Al2O3 and SiO2) at 1550°C using ImageJ**. **(a)** original particle image and **(b)** the manually tracked particle using ImageJ (yellow line).

**
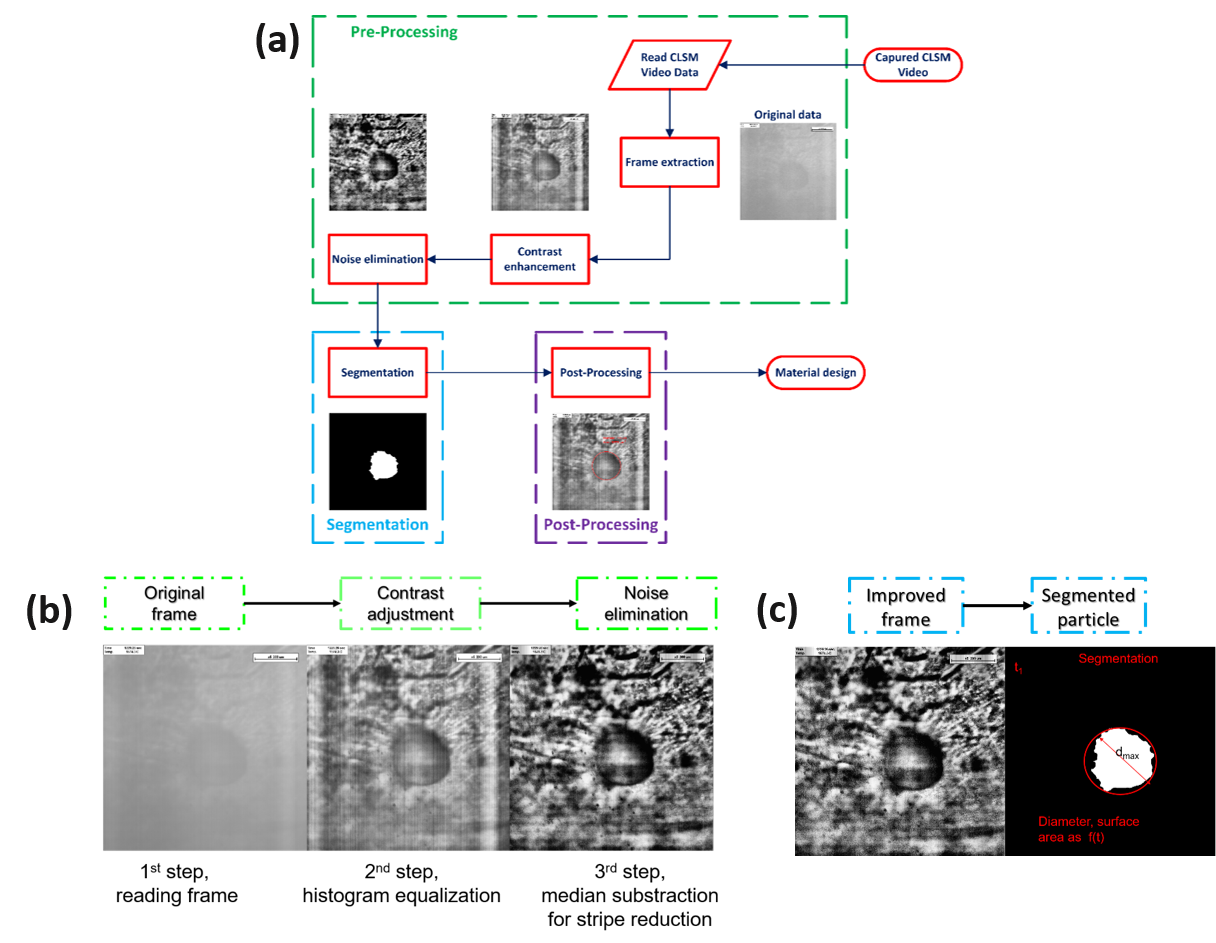
**

**Supplementary Figure 7|** **Dissolution particle evaluation using intensity-based segmentation technique**. **(a)** The workflow consists of three main components: pre-processing, segmentation, and post-processing. Pre-processing converts video into images, enhances contrast, and eliminates noise. Segmentation uses intensity-based thresholding to segment zirconia particles. Post-processing calculates the diameters of segmented particles. **(b)** two image quality enhancement steps are contrast enhancement (middle image) and stripe noise elimination (right image). **(c)** the equivalent diameter of an exemplary segmented particle is demonstrated.

**
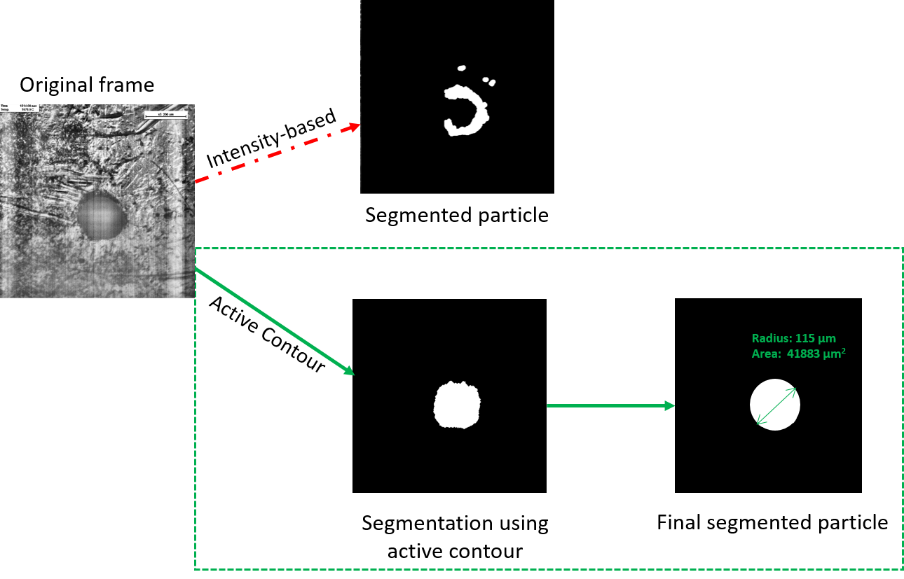
**

**Supplementary Figure 8|** **Comparison of intensity-based and active contour segmentation techniques.** The active contour demonstrated superiority in evaluating zirconia particle dissolution, as illustrated by the representative segmented particle obtained from two techniques.

**
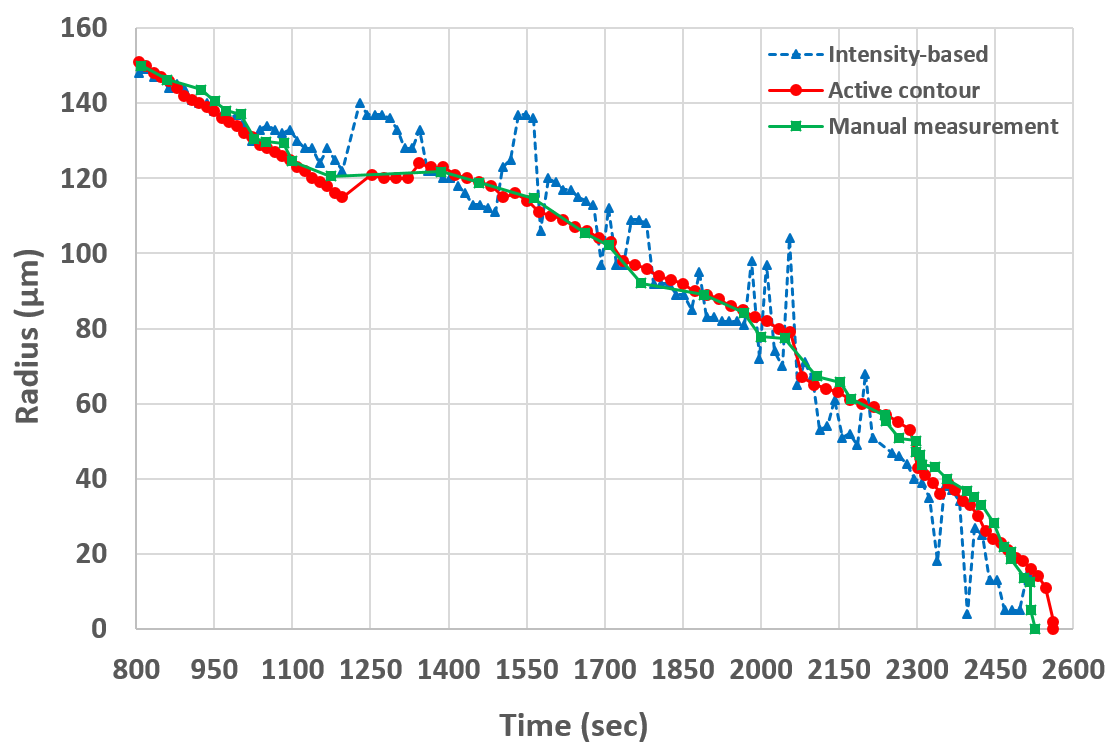
**

**Supplementary Figure 9| Radius evaluation of zirconia particle dissolution using two conventional segmentation techniques and a manual one.** The evaluated radius obtained from active contour and intensity-based segmentation is comparable to the manual evaluation.

**
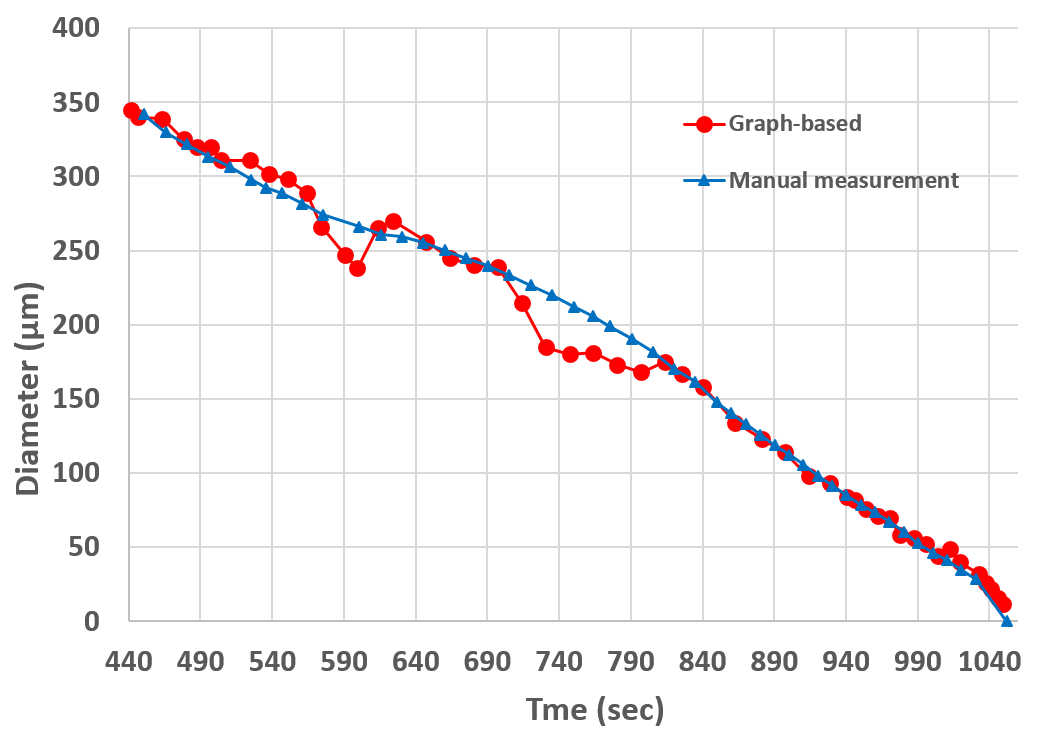
**

**Supplementary Figure 10|** **Comparison of calculated dissolving diameter of sapphire particles among graph-based segmentation techniques and manual particle evaluation.** The evaluated diameter obtained from graph-based segmentation is comparable to that obtained from manual evaluation.

**
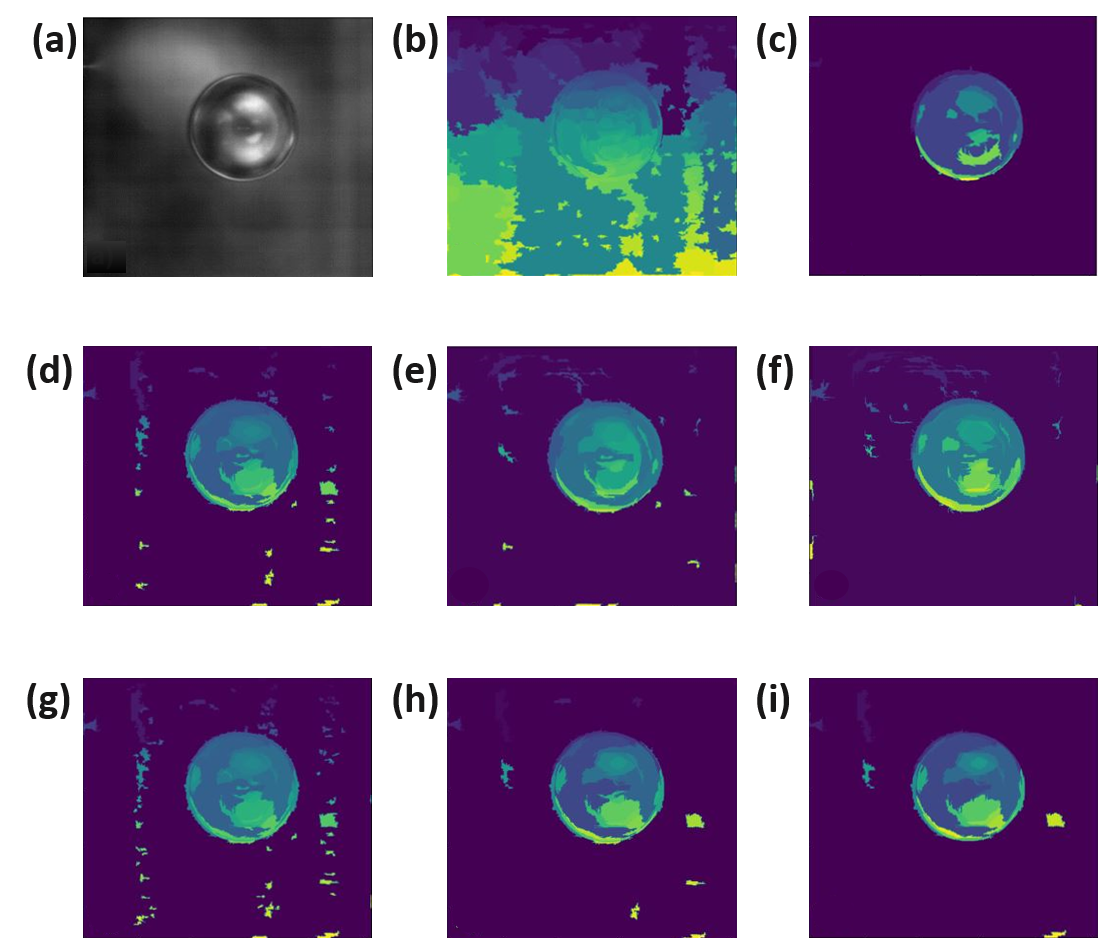
**

**Supplementary Figure 11|** **The impact of parameters setting in particle (sapphire) segmentation by the Felzenszwalb technique.** **(a)** shows the original frame and the segmentation results with the setting of the parameter scale to 10 and 70, as shown in **(b)** and **(c)**, respectively. The values of sigma and min_size is set to 1.1 and 200 in both setups. The particle segmentation by setting the values of sigma as 1.0, 1.5, and 2.0, while the values for scale = 30 and the min_size = 200 are shown in **(d)**, **(e)**, and **(f)**, respectively. In **(g)**, **(h)**, and **(i)**, the impact of different values of min_size equal to 100, 550, and 1000 are shown correspondingly. However, the values of scale and sigma are fixed at 30 and 1.0, respectively.

**
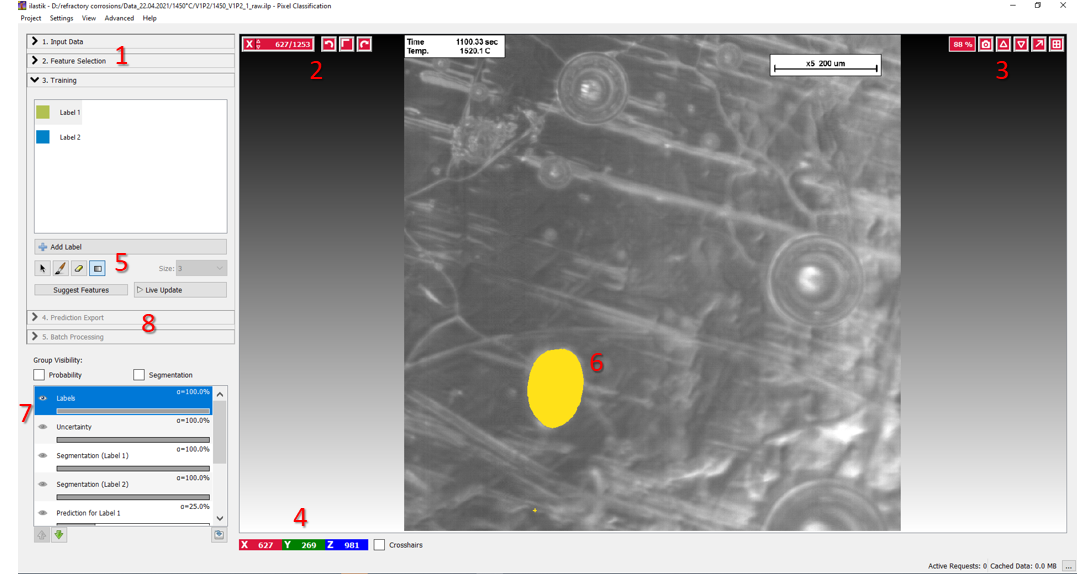
**

**Supplementary Figure 12|** **The user interface of Ilastik with an exemplary labelled frame from a HT-CLSM video at 1450°C.**

**
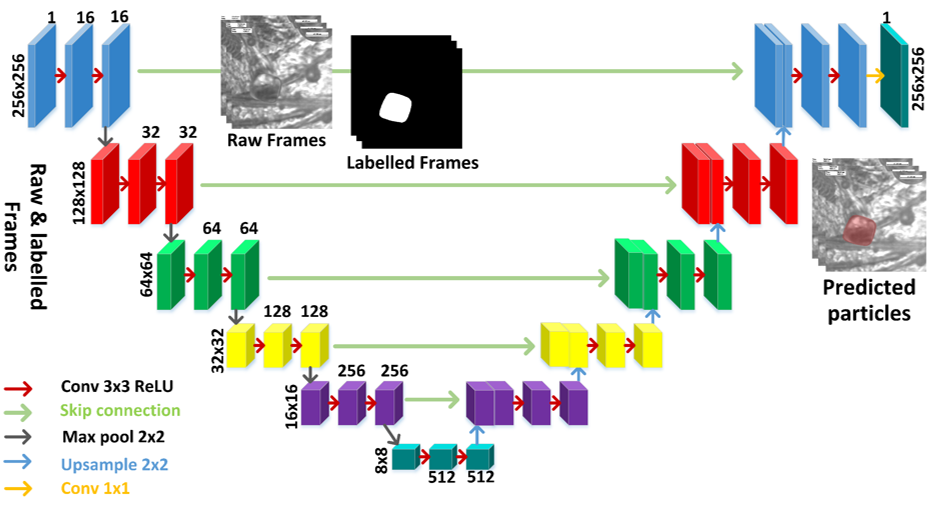
**

**Supplementary Figure 13|** **Architecture of the developed U-Net model.**

**SUPPLEMENTARY NOTES**

**Supplementary Note 1: Manual dissolving particle quantification**

The software ImageJ is used for the manual particle evaluation. It is an open-source image processing and analysis software based on the Java programming language. There are several drawing tools and built-in functions available for tracking and evaluating the dissolving particle. The first step is to set the scale based on the magnification of captured video from HT-CLSM experiments. Then manually specifying the particle boundary using the drawing tools of ImageJ (**Supplementary Figure 6b**). The built-in function measurement automatically returns the area of the localized particle surface. The equivalent diameter of the particle is calculated from the measured area (yellow circle in **Supplementary Figure 6b**) according to the equation (1).

$Equ. Dia=\sqrt[2]{\frac{measured area \times4}{\pi}}$ (1)

**Supplementary Note 2: U-Net architecture**

**Supplementary Figure 6b** shows the architecture of the developed U-Net, which includes the encoder and decoder paths that are connected by a bottleneck or bridge. The architecture of the encoder path is based on the common convolutional network. In each step of the encoder path, there are two convolutions with a 3x3 kernel size, a rectified linear unit (ReLU) as an activation, and the same padding to cover the boundaries of the input image and have the same output dimension. After the first convolution, there is a dropout layer for avoiding overfitting, and following the second convolution, the pooling layer (max pooling with 2x2 operation) is responsible for down-sampling (reduction of the spatial dimension and doubling the feature map). The middle layer (after the encoder path) consists of the two convolution layers and a dropout layer with the same settings as the encoder path. In the decoder path, every step comprises one transposed convolution layer (with a kernel size of 2x2, and the same padding) for up-sampling, followed by a skip connection. The long skip connection combines or concatenates the feature map from the transposed layer with the associated one from the encoder path in order to recover spatial information lost during down-sampling. The rest of the steps are similar to the encoder path, which includes two convolutions and a dropout in between. The final layer is a 1x1 convolution with a sigmoid as an activation.

We trained the model in similar steps as the atU-Net on the image size of 256x256.

The trained U-Net model was applied to all five videos of three temperatures, and the same investigations as atU-Net have been done on the predicted particles. Supplementary Figures 2, 3, and 4 illustrate the prediction accuracy of U-Net and indicate good alignment with manual prediction. **Supplementary Figure 5** shows the comparison among the prediction performance of both models, and the elliptical colored-dashes around the predicted dimeters’ over time in the plots show the superiority of atU-Net in accurate prediction of the particles.

**Supplementary Note 3: Intensity-based and active contour segmentation techniques**

As shown in **Supplementary Figure 7a,** the zirconia dissolving particle evaluation using the intensity-based technique was performed through three main steps. First of all, the CLSM video was read and converted to particle images (frames), and then the image quality was enhanced in two steps. Initially, the contrast was optimized using adaptive histogram equalization (**Supplementary Figure 7b** middle image). To eliminate the vertical stripes from every single frame, the median intensity of each column was calculated and subtracted from its corresponding minimum to provide a column offset. Afterward, bitwise subtraction of each pixel intensity and related column offset resulted in a noise-eliminated frame (**Supplementary Figure 7b** right image). To get the particle distinguished (segmented) from slag, a circular mask in the approximate location of the particle was put on each frame, and the calculated threshold using the histogram-based global thresholding method ^1^ was applied to the masked frame. The next step is to utilize the morphological operation of erosion to optimize the segmentation error. The equivalent diameter of segmented particle was calculated from binarized frame (**Supplementary Figure 7c**). Although the intensity-based technique facilitated the particle evaluation process, it failed to accurately segment particles in some frames due to a lack of contrast. The active contour segmentation technique was used to optimize these segmentation failures (**Supplementary Figure 8**). The same procedure as the previous technique was performed except for one step. The active contour technique using a circular mask with the Chan-Vese method was applied to segment particles instead of threshold in the intensity-based technique. **Supplementary Figure 9** shows that particle dissolution radius obtained from the active contour technique are comparable to the manual evaluation. These analyses was done in MATLAB R2018a.

**Supplementary Note 4: Graph-based segmentation technique**

An efficient graph-based image segmentation algorithm was developed by Pedro F. Felzenszwalb and Daniel P. Huttenlocher ^2^. This technique belongs to the minimum spanning tree (MST) method and is very computationally efficient while capturing certain relevant non-local image characteristics. Due to these properties, it is widely used and is also able to run at video rates. This technique is suitable for segmenting of the dissolution of sapphire particles in different slags. The main difference between the zirconia and sapphire particles is their transparency and moving behavior. While the zirconia particle is not transparent and remains mostly in the same place, the sapphire particle is moving around in the crucible and may be transparent. Due to these specifications, the segmentation of sapphire particles is much more complicated than that of zirconia particles. The particle movement results in the background changing and the frames' blurriness. There are three parameters (i.e., scale, sigma, and min_size) that need to be set very carefully using this technique. The scale declares the observation level; the higher it is, the larger the clusters (**Supplementary Figure 11c**). The parameter sigma is the width of the Gaussian kernel used in preprocessing. The minimum size of components is controlled by min_size. The impact of two different values of the parameter scale is shown in **Supplementary Figure 11b, c**, while the other parameters are kept constant. By increasing the sigma, the segmentation will be smoother, as depicted in **Supplementary Figure** 11d to S11f. However, **Supplementary Figure 11i** shows that the bigger value of min_size resulted in a bigger cluster size. Once the parameter tuning is satisfactory, then the segmented clusters belonging to particles should be kept, and the undesired ones need to be deleted based on their labelled numbers. This procedure should be repeated for each image data (frame).

**Supplementary Note 5: Data labelling using Ilastik**

Ilastik ^3^ is a free and user-friendly open-source image-classification and -segmentation software. It provides various workflows for a wide range of applications, such as automated (supervised) pixel or object-level classification. Due to the complexity of the data in the current study, the Ilastik built-in classifiers failed for particle dissolution segmentation. However, it provides us with a suitable tool for data labelling (annotation). This manually labelled data was used as a part of the training set to train the developed U-Net model.

For a better understanding, the Ilastik user interface is shown in **Supplementary Figure 12**, and the application of the pixel classification workflow for manual labelling is described in detail as follows:

As a first step, a project is created based on the pixel classification workflow. After that, the raw data must be loaded using the option "Input Data" from the marked section "1" in **Supplementary Figure 12**. "Feature Selection" is the second option in this section that offers us a list of features (including color/intensity, edge, and texture). Each of these features can be selected on different scales of the sigma of the Gaussian smoothing. For labelling purpose, the selection of only one feature can be sufficient for the activation of the "Training" option in Section "1". In the "Training" option, two different labels with different colors are initiated, but there is the option to add or delete label(s).

The coordination system of Ilastik is shown in the area under Section "4" (**Supplementary Figure 12**). It is important to note that, this is different from our defined coordinate system. The y-axis in Ilastik corresponds to the width of the image, i.e., the x-axis of our coordinate system. The z-axis in Ilastik corresponds to the height, i.e., our y-axis. The x-axis is in accordance with the number of frames. The options above section "2" give us the possibility to access different frames, rotate the frames clockwise/un-clockwise, or swap them. The possibilities to export the current view of frame, zoom to fit, reset the zoom, dock/undock, and maximize the current view are given by the available options in the section above "3". The manual labelling can be mostly done using the brush and eraser options in the section "5". The contrast of the frames can be adjusted using the square option in this section. On the right side of this section, the size of the brush or eraser can be set. The option "Live Update" is used to train the classifier, and the prediction results will be overlaid on related images (this option is not used in data labelling).

A manual labelled particle is shown in the section "6". To do the labelling, we need to select the desired label and then paint the surface area of the particle very precisely, even at pixel level, especially at particle edges. The prepared labelled data can be exported by right-clicking on the "labels" option in the section "7". The visibility of the data and overlaid label can be controlled using the eye option in this section.

The two options "Prediction export" and "Batch processing" in section "8" can be used for exporting the predicted data obtained from model training, and the latter option is for applying the pre-trained classifier on unknown images. These options are not applicable to our work.

**References**

1. Vorauer, T. *et al.* Multi-scale quantification and modeling of aged nanostructured silicon-based composite anodes. *Commun. Chem.* **3**, 141 (2020).

2. Felzenszwalb, P. F. & Huttenlocher, D. P. Efficient Graph-Based Image Segmentation. *Int. J. Comput. Vis.* **59**, 167–181 (2004).

3. Berg, S. *et al.* ilastik: interactive machine learning for (bio)image analysis. *Nat. Methods* **16**, 1226–1232 (2019).
